# 2511. Screening for Hepatitis B Virus (HBV) and Tuberculosis (TB) Infection among Non-U.S.-Born Adults from High HBV and TB Burden Countries

**DOI:** 10.1093/ofid/ofad500.2129

**Published:** 2023-11-27

**Authors:** Jacek Skarbinski, Yuching Ni, Jenna M Wick, Amit S Chitnis, Amy L Krueger, Devan Jaganath, Robert J Wong, Nicole Halmer

**Affiliations:** Kaiser Permanente Northern California, Oakland, California; Kaiser Permanente Division of Research, Oakland, California; Kaiser Permanente Oakland Internal Medicine Residency Program, Oakland, California; Alameda County Public Health Department, San Leandro, California; CDC, Atlanta, Georgia; University of California, San Francisco, San Francisco, California; Stanford Medicine, Stanford, California; Kaiser Permanente - Division of Research, Oakland, California

## Abstract

**Background:**

Hepatitis B virus (HBV) infection and tuberculosis (TB) infection both disproportionately affect non-U.S.–born persons from countries with hepatitis B surface antigen (HBsAg) seroprevalence ≥2% or TB incidence rates ≥20/100,000 population. Early identification and treatment of chronic HBV and TB infection are critical to reduce morbidity and mortality, but little is known about HBV and TB screening among non-U.S.–born persons in the United States.

**Methods:**

In a cross-sectional study using electronic health record data, we assessed HBV and TB infection screening practices among Kaiser Permanente Northern California eligible members aged ≥18 years born in countries with intermediate to high burdens of both HBV and TB infection. HBV screening was defined as testing for HBV surface antigen and HBV core antibody. TB infection screening was defined as testing with interferon gamma release assay or tuberculin skin test. We calculated the proportion of individuals from intermediate and high HBV-TB burden countries who had HBV or TB infection screening and the proportion of patients positive for HBV or TB infection.

**Results:**

Of 498,339 non-U.S.–born adults, 275,708 (55.3%) were born in intermediate or high HBV-TB burden countries. Most patients were aged ≥50 years (65.3%) and 23.5% had diabetes. Among persons born in intermediate or high HBV-TB burden countries, 67.1% were screened for HBV infection, 29.4% were screened for TB infection, and only 23.7% were screened for both HBV and TB infection. Among all persons born in intermediate or high HBV-TB burden countries (both screened and unscreened), 3.7% had HBV infection and 10.1% had TB infection. Among 231,431 persons born in intermediate or high HBV-TB burden countries without current or prior HBV infection, 72.9% had received at least one HBV vaccine dose.

**Conclusion:**

Among non-U.S.–born adults from intermediate or high HBV-TB burden countries, we found low screening proportions, intermediate prevalence of chronic HBV infection and high prevalence of TB infection. Given the large population of persons from intermediate or high HBV-TB burden countries in Kaiser Permanente Northern California, screening for both infections at the same time could improve early diagnosis and treatment for both conditions.

**Disclosures:**

**Jacek Skarbinski, MD**, Genentech: Grant/Research Support|Gilead Sciences: Grant/Research Support **Robert J. Wong, MD, MS**, Gilead Sciences: Grant/Research Support

